# P-1489. Evaluating the public health impact of infant RSV prophylaxis programs in Spain and the UK: results from the ongoing REACH study

**DOI:** 10.1093/ofid/ofaf695.1673

**Published:** 2026-01-11

**Authors:** Oliver Martyn, Rolf Kramer, Karine Mari, Esther Donkers, J Jasper Deuring, Saul N Faust

**Affiliations:** Sanofi Vaccines, Copenhagen, Hovedstaden, Denmark; Sanofi Vaccines, Lyon, France, Lyon, Rhone-Alpes, France; Sanofi Pasteur S.A., Lyon, Auvergne, France; LOGEX, Amsterdam, Noord-Holland, Netherlands; LOGEX, Amsterdam, Noord-Holland, Netherlands; University of Southampton and University Hospital Southampton NHS Foundation Trust, Southampton, England, United Kingdom

## Abstract

**Background:**

Respiratory syncytial virus (RSV) is a leading cause of infant respiratory infections. The UK has implemented maternal vaccine to prevent RSV in infants from autumn 2024/5 whereas Spain implemented nirsevimab monoclonal antibody treatment in 2023/4 and 2024/5. The REACH study aimed to quantify the reduction in burden amongst infants of RSV-related hospital care across Spain and the United Kingdom (UK).

**Figure 1:**
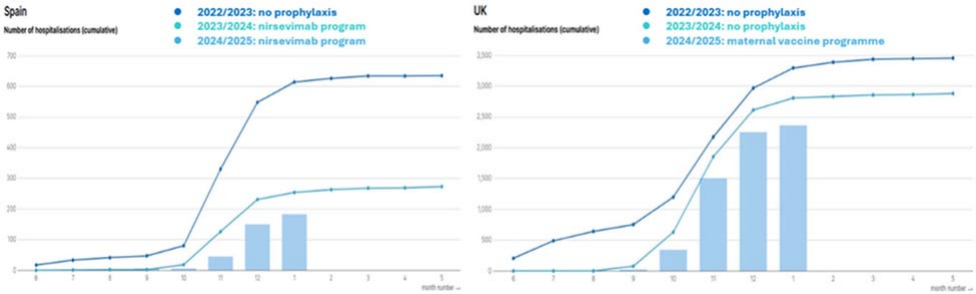
Cumulative RSV hospitalizations in infants <12 months of age (ICD-10 coded) up to 31-Jan-2025

**Methods:**

This observational, retrospective, multi-center cohort study utilizes administrative and microbiology data from hospital sites in Spain and the UK, covering three consecutive RSV seasons from June 2022 to May 2025. Results are stratified by country, RSV season, patient risk groups, and age groups. The primary objective is to report by country the number of ICD-10 coded lower respiratory tract infection (LRTI) and RSV-LRTI hospital admissions, length of stay (LOS), and ICU admissions. Additional objectives include reporting laboratory-confirmed RSV-positive LRTI hospitalizations and estimating direct hospitalization costs, HCRU in the 30-day period post-discharge and concordance between ICD-10 and laboratory-test based diagnoses.

**Results:**

Amongst infants < 12 months of age, the cumulative number of RSV hospitalizations, based on ICD-10 coded data, was reduced by 70.2% in Spain and 28.3% in the UK compared to the 22/23 (pre-prophylaxis) season, up until latest data cut-off (31-Jan-2022 vs. 31-Jan-2025). Amongst infants < 6 months of age, the reduction was 78.1% in Spain and 32.5% in UK. Amongst infants aged 13-24 months who did not benefit from prophylaxis, a similar trend with minimal change was observed in both countries, suggesting normal RSV circulation.

**Conclusion:**

Our preliminary results suggest substantial differences in the public health impact of the respective immunization campaigns targeting infants and pregnant women in Spain and UK. Data collection is ongoing, and full season results until end of May 2025 are expected by the time of ID week.

Funding: Sanofi and AstraZeneca

**Disclosures:**

Oliver Martyn, MPH, Sanofi: Employee Rolf Kramer, PhD, Sanofi: Employee|Sanofi: Stocks/Bonds (Private Company) Karine Mari, MSc, Sanofi: Employee J. Jasper Deuring, PhD, Pfizer: Stocks/Bonds (Private Company) Saul N. Faust, FRCPCH PhD, AstraZeneca: Advisor/Consultant|AstraZeneca: Clinical trial investigator on behalf of institution|BioNTech SE: Funding of clinical trial to institution|CSL Seqirus: Advisor/Consultant|GSK: Clinical trial investigator on behalf of institution|J&J: Advisor/Consultant|J&J: Clinical trial investigator on behalf of institution|MedImmune: Advisor/Consultant|Merck: Clinical trial investigator on behalf of institution|Moderna: Honoraria|Moderna: Clinical trial investigator on behalf of institution|MSD: Advisor/Consultant|Novavax: Honoraria|Pfizer: Advisor/Consultant|Pfizer: Honoraria|Pfizer: Clinical trial investigator on behalf of institution|Sanofi: Advisor/Consultant|Sanofi: Clinical trial investigator on behalf of institution|Valneva: Clinical trial investigator on behalf of institution

